# Anticancer action of naturally occurring emodin for the controlling of cervical cancer

**DOI:** 10.37349/etat.2023.00161

**Published:** 2023-08-31

**Authors:** Priyanka S. Lande, Vaibhav S. Adhao, Jaya P. Ambhore, Kiran P. Gaikwad, Chanchal S. Chandak, Leena P. Joge

**Affiliations:** NGO Praeventio, Estonia; Department of Quality Assurance, Dr. Rajendra Gode College of Pharmacy, Malkapur, Dist-Buldhana 443101, Maharashtra, India

**Keywords:** Anticancer, emodin, anti-proliferative, anti-carcinogenic

## Abstract

One of the major causes of death on the globe is cancer. The fourth most frequent malignancy in women worldwide is cervical cancer. Several cancer patients are remaining incurable due to the emergence of medication resistance, despite notable advances in cancer research over the previous few decades. The importance of natural sources as possible therapeutic candidates may be significant. Anthraquinones are one of the many chemical families of natural products, and they stand out for their wide range of structural variations, notable biological activity, and low toxicity. A natural substance called emodin, an anthraquinone derivative, is present in the roots and rhizomes of several plants. This substance has demonstrated antineoplastic, anti-inflammatory, antiangiogenic, and antiproliferative properties. It is also capable of preventing cancer spread and can reverse cancer cells’ multidrug resistance. Emodin, a broad-spectrum inhibitor of cancer cells, have anticancer properties in many different types of biological pathways. These molecular mechanisms in cancer cells include the suppression of cell growth and proliferation, deterioration of the cell cycle arrest, the start of apoptosis, antimetastasis, and antiangiogenic impact. Therefore, the aim of the present review summarised the antiproliferative and anticarcinogenic qualities of cervical cancer of emodin.

## Introduction

The biggest health concern in the world is cancer. A rise in cancer incidence, primarily brought on by aging populations, is shown by high morbidity and fatality rates. Abnormal vaginal bleeding, vaginal discharge, pelvic pain, or pains during sexual activity are all signs of cervical cancer [1]. Among women around the world, cervical cancer is the fourth most frequently found cancer. India is said to be responsible for 27% of all cervical cancer fatalities, with developing nations accounting for 85% of cases and deaths from the disease [2]. Human papillomavirus (HPV) infection is connected to it. A significant proportion of female deaths are still linked to cervical cancer [3], despite efforts to prevent HPV infections through vaccination, since vaccinations may offer cross-protection against some HPV strains that cause cervical cancer. The most popular treatments for cervical cancer include surgery, including radical hysterectomy and pelvic lymphadenectomy, radiation therapy, and chemotherapy [4]. Another treatment for cervical cancer is targeted therapy, which inhibits the cyclooxygenase-2 (COX-2) and epidermal growth factor receptor (EGFR) [5, 6]. However, these therapies could have negative side effects and complications. For instance, surgery could cause major bleeding, organ damage, and the danger of blood clots in the deep veins of the legs. Radiotherapy could also cause menopause, infertility, discomfort, or pain during sexual activity. Moreover, several side effects and treatment resistance are associated with the drugs frequently used to treat cervical cancer [7–11]. One of the most effective anticancer drugs, cisplatin, can build resistance via a defense mechanism [12]. Patients with cervical cancer have also reported 5-fluorouracil (5-FU) resistance and negative consequences [13]. So, the present study has thus focused on creating a potent new treatment for cervical cancer using natural ingredients.

Emodin (1,3,8-trihydroxy-6-methyl-anthraquinone), a natural anthraquinone derivative, is mostly found in the rhizomes and roots of Rhamnaceae, Polygonaceae, Rubiaceae, and Fabaceae plants. It shares chemical similarities with the anthracycline core utilized in cancer therapies that involve anthracyclines (carminomycin, daunorubicin, nogalamycin, and doxorubicin). Emodin has a wide range of properties, including those that are immunomodulatory, antibacterial, and anti-inflammatory. On several cancerous cells, emodin also exerts cytotoxic and growth-inhibitory actions. In addition to helping with metabolic and immune functions, it acts as a vasomotor system modulator [14]. These substances are also notable for their capacity to bind with DNA and restrain the activity of the enzyme topoisomerase-II (topo-II). By stabilizing topo-II-DNA covalent complexes and inducing apoptosis in cells, emodin damages DNA [15].

## Chemical nature of emodin

Anthraquinones, as, specialized metabolites along an anthracene moiety involving 3 benzene rings along with a ketone group in positions nine (9) and ten (10) as an essential skeleton as well as substitution with different variety of efficient groups such as -CH_3_, -OH, -CH_2_OH, -OCH_3_, -CHO, -COOH, also their products. The anthraquinone fragments that hold the hydroxy group are defined as hydroxyanthraquinones. 1,8-Dihydroxyanthraquinones substituted with the hydroxy groups from the C-1 carbon and C-8 carbon show acidic nature which is equivalent to that of the carboxyl group then they have related arrangements. They can originate as glycosides in nature as with their free aglycone form but they are typically joined to sugar molecules like glucose, rhamnose, glucorhamnose, or primevalerose [16, 17].

**Figure 1:**
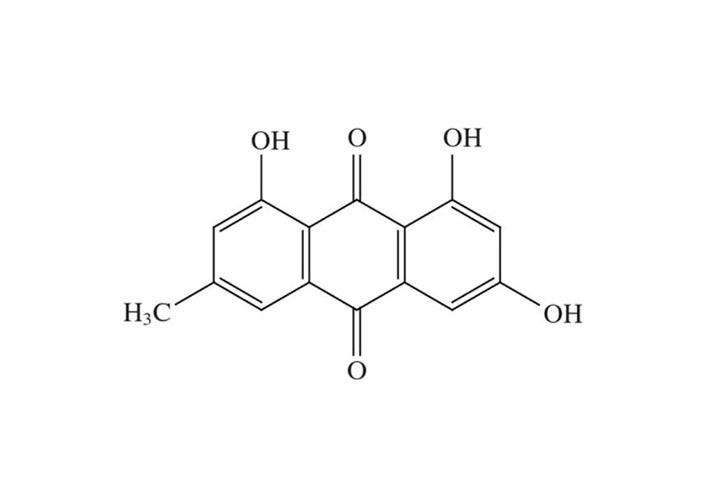
Structure of emodin

The biological anthraquinone emodin, also known by its chemical name 1,3,8-trihydroxy-6-methyl anthraquinone (Figure 1), is found in traditional medicinal herbs. Emodin is typically observed in roots, radix, and rhizomes of rhizome [18]. Numerous features of emodin, including anticancer efficacy and cytotoxicity against cervical carcinoma cells associated with high-risk HPV, have been demonstrated [19, 20].

## Anticancer activity of emodin for the management of cervical cancer

Bu 25TK, Ca Ski, HeLa, and ME-180 cells were used in the initial investigation to examine emodin’s impact on cervical cancer. Treatment with emodin (50 µmol/L) demonstrated some apoptotic changes in cell lines. The most sensitive cell, Bu 25TK, exhibited a lethal dose 50 (LD_50_) of 56.7 µmol/L and was greatly inhibited in its ability to proliferate by emodin in a concentration-dependent manner. It has been demonstrated that emodin causes DNA damage, nuclear condensation, phosphatidyl serine translocation to the outer membrane surface, and poly[adenosine 5’-diphospho adenosine diphosphate (ADP)-ribose] polymerase cleavage. Caspases 3 and 9 were active; however, caspase 8 activities remained constant in Bu 25TK cells [21].

As reactive oxygen species (ROS) play a significant role in arsenic trioxide-induced apoptosis, Yang et al. [22] investigated emodin’s potential to increase cellular ROS levels in HeLa cells and its capacity to boost apoptotic sensitivity to arsenic trioxide. The results suggest that emodin may accelerate arsenic-induced apoptosis by generating ROS at a concentration of 10 µmol/L while posing no risk to healthy fibroblast cells. The inhibition of nuclear factor-kappaB (NF-κB) activation and a reduction in mitochondrial transmembrane potential was linked to elevated ROS levels [22]. Cervical cancer cells HeLa cells cultured with aloe-emodin, causing cell cycle arrest in the G2/M phase. The cells revealed a reduction in cyclin A and cyclin-dependent kinase 2 (CDK2), indicating that proliferation and differentiation were inhibited, as well as inhibition of protein kinase C (PKC) and cellular Myc (c-MYC), indicating that proliferation and differentiation were suppressed. Increases in cyclin B1, CDK1, and alkaline phosphatase (ALP) activity was detected, together with suppression of proliferating cell nuclear antigen (PCNA), indicating reduced proliferation [23]. By preventing lipid raft coalescence, integrin aggregation, and focal adhesion complex (FAC) formation in human breast cancer (MDA-MB-231) and human cervical epithelioid carcinoma (HeLa), emodin prevents tumour cell attachment [24].

A different study used SiHa and C33A cervical cancer cells to show how emodin affects DNA damage and intracellular ROS levels [25]. Moreover, it inhibited Ak strain transforming (AKT) activation in both SiHa and C33A cells in the concentration range of 46.3–185.0 µmol/L, increased Bcl-2-associated X protein (Bax 2), and decreased B-cell lymphoma 2 (Bcl-2) expression [25].

Apoptosis and DNA damage were both shown to occur in studies by Liu et al. [26]. Emodin caused extensive genomic DNA damage and the death of HeLa cells when administered at doses ranging from 1 µmol/L to 10 µmol/L. At dosages ranging from 5 µ mol/L to 20 µmol/L, S-phase cell cycle arrest and telomere degradation were observed [26].

In HeLa cells treated with 40 µmol/L emodin, the suppression of casein kinase 2 (CK2) was unrelated to the downregulation of AKT kinase. The results with AKT1, AKT2, and AKT3 showed that emodin subdued upstream proteins that indicate to and up-regulate AKT rather than directly changing AKT activity. The catalytic activity of the mammalian target of rapamycin (mTOR) kinase was significantly reduced by 60 µmol/L emodin by lowering the amount of phosphorylation of the AKT protein. Moreover, there was a little decrease in the expression of the phosphatase and tensin homolog (PTEN) protein, but there was no change in the total protein levels of c-Jun N-terminal kinase (JNK), p38, or p44/2 MAP kinase [27].

Apoptosis was found to be 8.2–43.7% in a different study on HeLa cells where emodin caused morphological changes indicative of apoptosis depending on the dosage in the concentration range of 20 µmol/L to 80 µmol/L. After 48 h of emodin therapy, pro-caspase-9, pro-caspase-8, and pro-caspase-3 expressions reduced while that of caspase-9, caspase-8, and caspase-3, cytochrome c (Cyt c), and apoptotic protease activating factor 1 (Apaf-1) rose. The results of the tests demonstrated that emodin induces apoptosis in HeLa cells through both intrinsic and extrinsic death receptor mechanisms [28].

It has been investigated how emodin causes apoptosis in cervical cells by activating the transforming growth factor (TGF)-signaling pathway in SiHa and HeLa cells. By reducing the activation of phosphorylated Smad3 (p-Smad3), p-Smad4, and TGF-receptor II, as well as by interfering with TGF-induced migration and invasion, it has been demonstrated that 40 µmol/L emodin has a significant impact on the TGF-signalling system [29].

In Hela, JAR, and HO-8910 cells, emodin at doses of 5 µmol/L, 10 µmol/L, and 15 µmol/L reduced proliferation and obstructed migration. Increased matrix metalloproteinase-9 (MMP-9) mRNA expression demonstrated emodin’s efficacy in invasion prevention. All cells were arrested in the G0/G1 phase and there was DNA damage, an increase in caspase-9 and matching activation of cleaved caspase-3, a drop in M phase, and a decrease in Bcl-2 [30]. Emodin significantly altered the lysosomal compartment, elevated autophagic vacuoles, and boosted lysosomal hydrolase activity in HeLa cells at doses between 1 µmol/L and 100 µmol/L [31].

In a recent study, Trybus et al. [32] focused on the apoptotic mechanism of emodin in HeLa cells at doses ranging from 1–100 µmol/L. The results showed that it slowed cell division most severely at a concentration of 100 µmol/L, induced cell cycle arrest in the G2/M phase, increased the frequency of cells going through mitotic catastrophe, and enhanced cytoskeleton changes [32].

Studies show that emodin damages DNA increases ROS levels, and lowers the potential of the mitochondrial membrane even though cervical cancer is the second most common malignancy in the world in terms of incidence and death [33]. In several cervical cancer cell lines, emodin suppressed the cell cycle in the S, G0/G1, and G2/M phases and stopped the cell cycle in the S, G0/G1, and G2/M phases. Several studies have shown the reduction of mTOR kinase catalytic activity, activation of caspase-3 and -9, and down-regulation of AKT kinase [33]. Based on the results, additional research to fully understand the effectiveness of emodin in the treatment of colon cancer would surely be helpful. Moreover, aloe-emodin causes G2/M arrest *in vitro* in human oral cancer KB cells [34], human promyelocytic leukaemia HL-60 cells [35], and cervical cancer HeLa cells.

**Figure 2:**
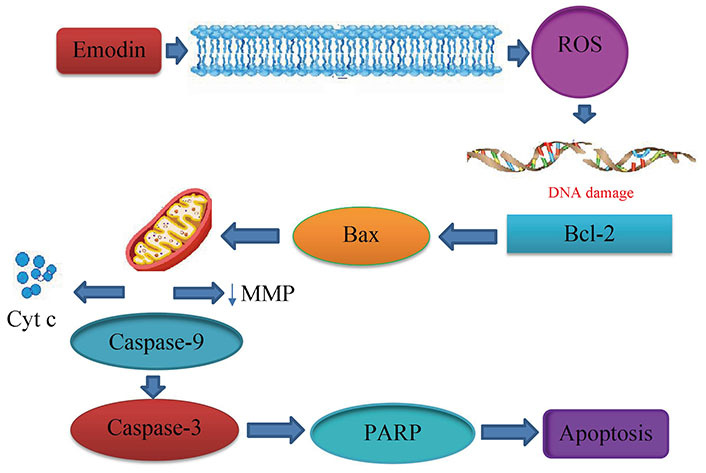
Anticervical apoptosis mechanism. PARP: poly(ADP-ribose) polymerase; ↓: lowers the matrix metalloproteinase

**Figure 3:**
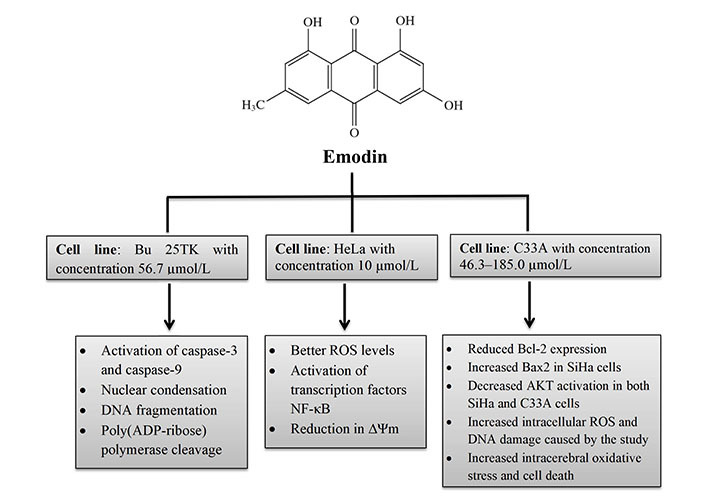
*In vitro* biochemical effects of emodin in cervical cancer cell lines Bu 25TK, HeLa, and C33A. ∆Ψm: mitochondrial membrane potential

**Figure 4:**
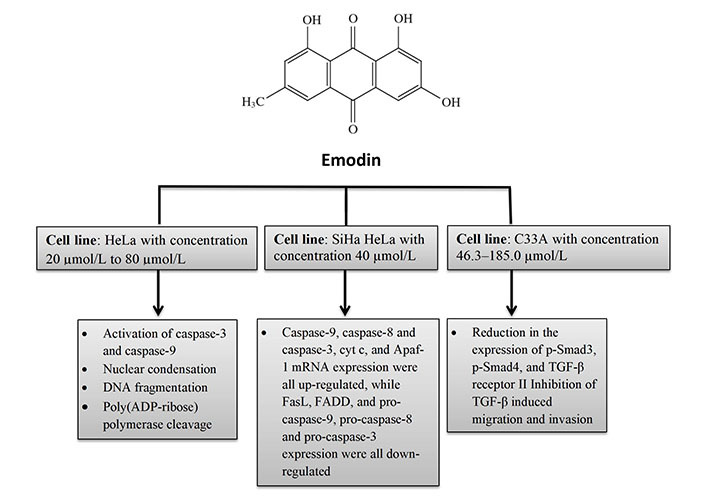
*In vitro* biochemical effects of emodin in cervical cancer cell lines HeLa, SiHa HeLa, and C33A. FasL: Fas ligand; FADD: Fas-associated protein with death domain

Aloe-emodin was employed to treat cervical cancer cells (HeLa), which led to cell cycle arrest in the G2/M phase. The cells displayed decreased levels of cyclin A and CDK2, which inhibit cell proliferation, as well as PKC and c-MYC, which indicate that differentiation and proliferation were, inhibited [36]. PCNA was inhibited coupled with increases in cyclin B1, CDK1, and ALP activity, indicating reduced proliferation. Emodin promotes apoptosis, causes cell cycle arrest, modifies immunological signalling, and alters cell motility in many cancer cell lines. Via intrinsic and extrinsic (Cyt c/caspase 9) apoptotic mechanisms, emodin reduces the survival of cancer cells. These pathways are linked to negative effects on mitochondrial membrane permeability and/or oxidative stress via increased ROS production. The anti-cervical apoptosis pathway is shown in Figure 2. *In vitro*, the biochemical effects of emodin in cervical cancer cell lines are summarized in Figure 3 and Figure 4.

## Anticancer activity of emodin for the management of various cancers

Yu et al. [37] describe their research associated with the emodin anticancer effect utilizing the human cell lines LNCaP, DU-145, and PC-3. They found that emodin triggered cell death in PC-3 cells via the mitochondrial pathway and reduced LNCaP cell growth via the androgen receptor and p53-p21 pathways [37]. Ok et al. [38] research on the human cell lines LNCaP, DU-145, and PC-3 to carry out the anticancer outcome of emodin reveals that CXC chemokine receptor-4 (CXCR4) and HER2 expression were decreased without impacting the viability of prostate and lung cancer cells [38].

Li et al. [39] usage of MCF-7 (human cells) to observe anticancer efficacy against breast cancer results in the inhibition of cell proliferation at half maximal inhibitory concentration (IC_50_) of 90.2 µmol/L. The modification of apoptosis-related genes’ expression led to inhibition and apoptosis [39]. Using MCF-7 and MDA-MB-453 (human cells), Huang et al. [40] test the anticancer impact of emodin to decreased endoplasmic reticulum (ER) protein levels and suppressed ER transcriptional activation, which in turn restricted cell growth 20 µmol/L IC_50_ for toxicity against MCF-7 cells [40]. The proliferation of SKBR3 (human cells) was checked by Kalkhoran et al. [41] and work with an IC_50_ value of 25 µmol/L raised the expression of the mRNAs for caspase-3, caspase-8, caspase-9, and Bax, decreased the expression of Bcl-2, and induced apoptosis [41].

Li et al. [42] showed that emodin had anticancer action on A2780/taxol (human cells). The scientists noted that emodin caused apoptosis and increased susceptibility of cancer cells to paclitaxel by down-regulating glycoprotein, X-linked inhibitor of apoptosis protein (XIAP), and surviving [42].

Su et al. [43] tested the effectiveness of emodin against lung cancer cells using the human cell line H1299, and the results showed that emodin reduced viability and promoted apoptosis [43]. Emodin inhibited p53 protein aggregation, which can increase the level of autophagy in A549 lung cancer cells, but did not affect autophagy in healthy non-cancerous Ha CaT cells, according to research by Haque et al. [44].

Ma et al. [45] worked on cells T24 and T5637 (human cells) to check the emodin activity by the observation emodin suppresses the progression of bladder cancer by inhibiting the expression of Notch1 [45]. The anticancer mechanism of emodin against various cancers is concise in (Table 1).

**Table 1:** The anticancer mechanism of emodin against various cancers

**Emodin**	**Disease**	**Mechanism of action**
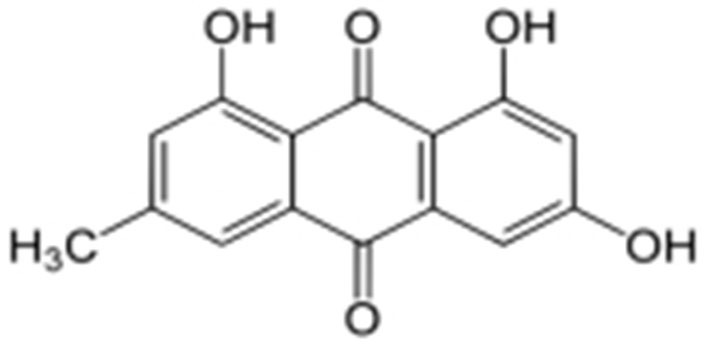	Cervical cancer	Inhibits mitotic activity Induces changes in the cytoskeleton, which induces mitotic death of cervical cancer cells
	Breast cancer	Inhibits the proliferation of MCF-7 human breast cancer cells Activation of aryl hydrocarbon receptor (AHR)
	Ovarian cancer	Inhibiting epithelial-to-mesenchymal transition (EMT) and ovarian cancer stem cells (CSC) formation
	Prostate cancer	Inhibition of multidrug resistance (MDR) and hypoxia-inducing factors
	Lung cancer	Inhibit the progression of lung cancer Affect the immune environment of the lung to treat lung cancer
	Bladder cancer	Emodin increased the cellular ROS level Enhanced the cisplatin-induced cytotoxicity of T24 and J82 human bladder cancer cells Decreasing glutathione-cisplatin (GSH-cisplatin) conjugates Blocked the chemoresistance of T24 and J82 cells to cisplatin by suppressing the expression of multidrug resistance-associated protein 1 (MRP1)

## Conclusions

Numerous investigations on the anticancer impact of bioactive chemicals have been conducted in recent years due to the rising incidence of cancer. Natural substances that can be derived from a variety of foods and plants may offer viable alternatives to current anticancer medications. The anticancer properties of anthraquinones against different cancer types are well recognized. The studies describing emodin anticancer activity against cervical cancer that have recently become available, either on its own or as a primary component of plant extracts, are compiled in this review. Emodin is one of the main elements of extracts derived from various medicinal properties. Plants, according to information gathered from the scientific literature. Numerous pharmacological studies have shown that emodin, an exceptional anti-cancer drug, offers a wide range of potential applications to treat various cancer hence as an outstanding antitumor agent therefore emodin is more potent to treat cervical cancer.

## Abbreviations

ADP: adenosine diphosphate
AKT: Ak strain transforming
Bax 2: B-cell lymphoma 2-associated X protein
Bcl-2: B-cell lymphoma 2
Cyt c: cytochrome c
CDK2: cyclin-dependent kinase 2
HPV: human papillomavirus
IC_50_: half maximal inhibitory concentration
ROS: reactive oxygen species
TGF: transforming growth factor

## Declarations

### Copyright

© The Author(s) 2023.
